# Physical Activity for Oncological Patients in COVID-19 Era: No Time to Relax

**DOI:** 10.1093/jncics/pkaa071

**Published:** 2020-08-24

**Authors:** Alice Avancini, Ilaria Trestini, Daniela Tregnago, Joachim Wiskemann, Massimo Lanza, Michele Milella, Sara Pilotto

**Affiliations:** 1 Department of Medicine, Biomedical, Clinical and Experimental Sciences, University of Verona, Verona, Italy; 2 Department of Oncology, University of Verona, Azienda Ospedaliera Universitaria Integrata, Verona, Italy; 3 Division of Medical Oncology, National Center for Tumor Diseases (NCT) Heidelberg and University Clinic Heidelberg, Heidelberg, Germany; 4 Neurosciences, Biomedicine and Movement Sciences, University of Verona, Verona, Italy

## Abstract

Whereas the coronavirus disease 2019 (COVID-19) storm is relentlessly progressing worldwide, a great effort from scientific societies has been made to give recommendations for safely continuing oncological care, prioritizing the interventions according to patients' condition and type and stage of tumor. Nevertheless, to date no specific suggestions regarding physical activity and exercise in cancer patients during the COVID-19 era have been released, neglecting the potential deleterious effects of quarantine and sedentary behaviour (imposed as containment measures against COVID-19), particularly in these subjects. Moreover, literature is constantly consolidating the crucial impact of regular physical activity in cancer in reducing recurrence and mortality risk. In this commentary, we discuss possible adaptations of the recently published exercise guidelines to the current pandemic emergency, proposing various modalities to prevent or mitigate the physical inactivity risk in cancer patients.

Since the new coronavirus disease 2019 (COVID-19), also known as SARS-CoV-2 (severe acute respiratory syndrome coronavirus 2) was announced in Wuhan in late December 2019, it has rapidly spread worldwide, prompting the World Health Organization to declare the pandemic on March 11, 2020 (1). Because of the high contagiousness and aggressiveness of this disease, on July 28, 2020, more than 16 341 920 cases and 650 805 related deaths have been reported around the world (1). COVID-19 patients can be completely asymptomatic (approximately 18% of cases) (2) or manifest several symptoms, ranging from mild to severe, mainly including respiratory manifestations (eg, rhinorrhea, sneezing, sore throat, cough, ground-glass opacities, pneumonia, hypoxemia, dyspnea, acute respiratory syndrome) and also systemic disorders (eg, fever, fatigue, headache, coagulation disorders, lymphopenia and other blood alterations, and gastrointestinal symptoms such as diarrhea and nausea) (3).

Preliminary data suggest that elderly patients (4) or those affected by chronic comorbidities (5) may be at higher risk of COVID-19 incidence occurring with a worse outcome (4,5). In particular, cancer patients seem to represent a high-risk category to experience COVID-19 disease with more severe manifestations, mainly due to compromised immune defenses and sequelae of antineoplastic treatments (5). Thus, given the current pandemic emergency, a great effort from scientific societies was performed to provide recommendations for safely continuing oncological care, prioritizing the interventions according to patients’ condition, type, and stage of tumor (6). Nevertheless, the emergency may unequivocally lead to postponing some anticancer treatments (5,6), further increasing patients’ anxiety and distress levels and therefore lowering compliance to therapy.

Considering that up to the time this paper was written, no vaccine or specific treatments against COVID-19 are available, the only way to keep the spread of the infection under control is the social distancing–that is, keeping people at home as much as possible, for as long as possible, until the COVID-19 outbreak is under control. Indeed, several countries around the world have adopted various containment measures (7). In Italy, for example, the national quarantine (ie, the prohibition for all people to move except for work, necessity, or medical needs) began on March 9, 2020, and it lasted until May 18, 2020, when a gradual reopening of commercial, productive, and social activities was allowed (8). Although these measures are strictly necessary, social distancing and quarantine may also have negative effects. A recent review has explored the impact of quarantine on psychological status, describing a high risk of posttraumatic stress symptoms, confusion, and anger (9). Moreover, this homestay period may lead to reduced physical activity (PA) and, thus, increased sedentary behaviors. In the general population, PA and sedentary time are respectively associated with positive and negative effects on body systems, mainly on muscle mass and cardiorespiratory fitness. Recent evidence highlighted the correlation between sedentary and risk of several chronic conditions such as metabolic syndrome, osteoporosis, cardiovascular and respiratory disease, stroke, cognitive function, and type 2 diabetes (10,11).

## Physical Activity and Exercise in Cancer

Despite the World Health Organization underlining the importance to be as active as possible during this quarantine period, it is reasonable to speculate that one of the groups that is decreasing its PA level is the oncological population. In this regard, patients usually reported a reduction in PA level after cancer diagnosis (12), with only approximately one-third of patients satisfying PA recommendations (13). In our experience, this proportion seems to be even smaller (14). Because of the current restrictions, this number could further diminish, amplifying the deleterious effects of sedentary behavior and physical inactivity.

PA is emerging as a key element in the oncological trajectory. A growing body of literature demonstrated the association between PA levels after a cancer diagnosis and survival (15). Collectively, these data reported a consistent, inverse correlation with all-cause mortality (ranging from 21% to 45%) and cancer-specific mortality (ranging from 26% to 69%) risk (15). Furthermore, some physical fitness components harbor a relevant impact in terms of both prognosis and recurrence risk. Cardiorespiratory fitness and muscular strength are prognostic factors in cancer patients (16). In addition, muscle-mass wasting has been connected with a worse treatment tolerance and higher risk of recurrence, overall, and cancer-specific mortality (17,18).

PA and exercise interventions are shown to be safe and feasible in oncological patients (19). A recent meta-analysis, including 48 randomized controlled trials with 3632 patients, found that exercise increases the peak of oxygen consumption by +2.80 mLO_2_*kg^-1^*min^-1^ in the interventional group compared with no changes in the control group (20). Padilha and colleagues (21) have investigated the role of resistance training or a combined exercise program (aerobic plus resistance) on muscle mass, strength, and body fat. The results have demonstrated that exercise was effective in improving muscular strength, regardless of the treatment type, concomitantly increasing lean body mass and decreasing body fat. Over the years, the role of PA and exercise as a strategy to improve health-related quality of life in cancer patients has been established (13,19). The improvement in quality of life could be partially associated with the efficacy of exercise in alleviating or preventing cancer- and treatment-related adverse events, such as cancer-related fatigue, lymphedema, anxiety and depression levels, bone health, and sleep quality, as well as cardiotoxicity risk, cognitive function, sexual function, chemotherapy-induced peripheral neuropathy, and nausea (19). Finally, limited data also exist regarding treatment tolerance (ie, the adherence to a planned therapy). In fact, exercise may improve the chemotherapy completion rate in patients physically active during adjuvant treatments compared with the control group (19).

## Efficacy of Home Exercise Programs in Oncology

The American College of Sports Medicine (ACSM) has released the new exercise guidelines for cancer survivors (19). ACSM suggests that an effective exercise prescription should include moderate-intensity aerobic training at least 3 times per week for 8-12 weeks. Moreover, the exercise program should add resistance-training activities, at least 2 times per week, using 2 sets of 8-15 repetitions at least 60% of 1 maximum repetition (19).

According to the current pandemic emergency, these guidelines should be adapted to a home-based setting because supervised sessions are not possible. A reliable solution can be represented by home-based exercise programs. The home-based exercise programs can exploit the telehealth (or telemedicine)—that is, the remote delivery of health care as well as a range of other services, including patient education and wellness promotion through technology (22). Telehealth programs do not have a formal structure to deliver information and can utilize different technologies, therefore allowing the exercise prescription and monitoring in several ways (22). For example, in cancer survivors, telephone counseling, short message services, digital media (eg, DVD), tailored and/or mailed materials, and/or computer and/or web-assisted programs were applied (23). Moreover, the home-based exercise programs are feasible, usually well accepted, and can be facilitated through the social support deriving from the patient’s family and the possibility to self-organize the free time, choosing when to perform the activities (24). If well structured, including, for example, an initial phase to educate patients (eg, to self-monitor the intensity), home-based programs have been demonstrated to be efficacious in improving lifestyle in cancer population. In this regard, Demark-Wahnefried and colleagues (25) have proposed a randomized trial, including 519 newly diagnosed breast and prostate cancer survivors, with the aim to improve diet and exercise practice using a tailored mail print intervention. The intervention included personalized workbooks followed by a series of newsletters (at 6-week intervals) that were tailored to barriers, stage of readiness, and progress toward goal attainment of exercising and nutritional aspects (25). Patients also received a survey on the current health practices and the willingness of starting and maintaining a lifestyle change, which was used to adapt the periodic newsletters (25). The study increased the weekly time spent in exercise, improved the overall diet quality and the daily intake of fruits and vegetables, and decreased fat intake and weight (25). In the recent years, thanks to the advent of technology in the PA context, a growing number of studies have tested different internet approaches for PA and exercise programs, such as a web-based system (26), mobile application (27), or social media (eg, Facebook) interventions (28), which found positive and meaningful results. Along these lines, a recent randomized trial tested a web-based exercise program in 68 breast cancer patients undergoing chemotherapy to determine the effectiveness in preventing the impairment of functional capacity, muscular strength, and anthropometric parameters, usually experimented during chemotherapy periods. The intervention consisted of an 8-week, web-based exercise program with 3 sessions per week, which were organized in warm-up, aerobic, and strength activities. The web system also permitted the communication between patients and research staff and weekly contacts with the aim to assure the correct performance and to tailor the program according to patients’ needs (26). The results demonstrated the intervention effectiveness in terms of both cardiorespiratory fitness and muscular strength, ameliorating the detrimental effects of treatments (26).

Nevertheless, the application of telehealth should be also considered outside the pandemic emergency. Because of the constant improvement in prevention, diagnosis, and treatments, the number of cancer patients and survivors is continuously increasing, and the financial resources available for supporting an exercise program could be limited. The home-based exercise program can offer a low-cost and sustainable alternative, especially when the costs are borne by the patients (29). In this regard, van Waart and colleagues (30), in a sample of 230 breast and colon cancer patients, evaluated the cost-utility and cost-effectiveness of 2 different PA programs compared with usual care. The home-based, low-intensity PA program costs €46 (approximately $53) per participant, whereas the moderate-high intensity, supervised exercise program costs €757 (approximately $849). Although the high willingness to pay may limit the cost-effectiveness of the home-based, low-intensity PA program (30), no definitive data are available in this sense.

Apart from the limited cost, telehealth offers the opportunity of easily spreading the access to exercise programs to cancer patients. For example, patients living in rural or remote communities are at high risk of being underserved in terms of health care and health-related services. Indeed, a recent study has reported that rural cancer survivors are 2.6 times less likely to meet aerobic PA guidelines than urban cancer survivors (31). This population should face the burden and discomfort of travel time to reach the services, thus decreasing the willingness to start a supervised exercise program. This statement is also confirmed by interesting data evaluating the exercise preferences in rural breast cancer survivors. Of the patients, 76% were interested in participating in an exercise program, the majority preferred to perform exercise at home (63%), and almost half (47%) of the participants favored an unsupervised program, endorsing the hypothesis that a remote exercise program could be well accepted by a rural cancer population (32). Telehealth can overcome these barriers and indirectly diminish the disparity in survival and disease-related outcomes existing between nonmetropolitan and metropolitan patients (33).

## Practical Considerations to Increase Exercise Level in Cancer Patients During COVID-19 Pandemic

**Figure 1.**
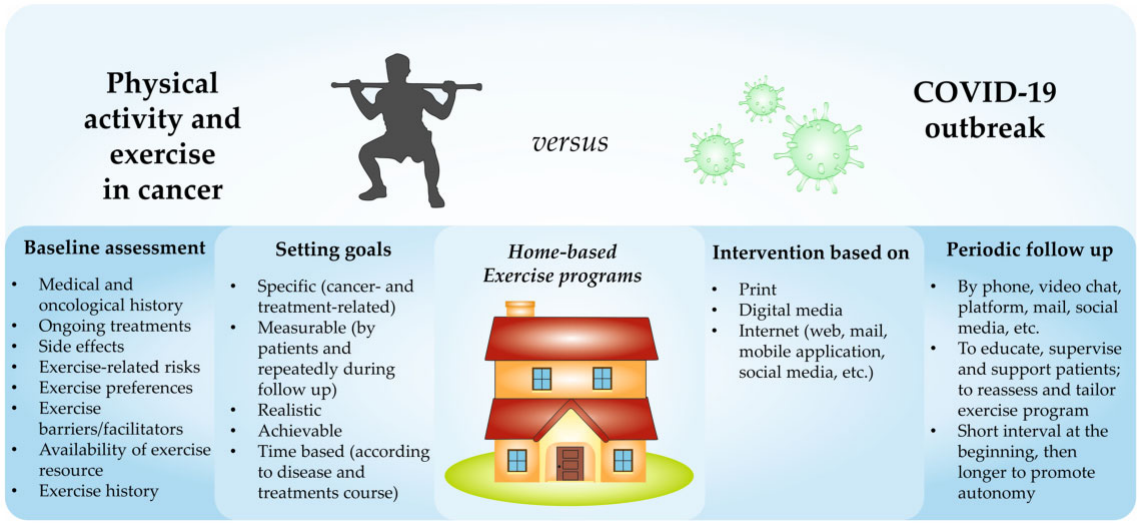
Proposed model of a home-based exercise intervention dedicated to cancer patients.

According to the aforementioned evidence and with the current containment measures, several modalities are available to support an effective home-based exercise intervention (Figure 1).

The COVID-19 outbreak makes it necessary to remotely perform all the steps of exercise prescription, which are usually carried out face to face. The health-related physical fitness can be hard to test in this framework. Nevertheless, an initial evaluation may be proposed at a distance through a videoconference, especially for those patients starting an exercise program (Figure 1). Ideally, this phase should include different assessments. On the one hand, patients’ health history (including cancer characteristics and comorbidities), current treatments, presence and severity of side effects, and screening tools to assess the exercise risk are essential to prescribe a safe program (19,34). On the other hand, understanding exercise preferences, barriers, facilitators, availability of resources to support exercise engagement, and patients’ exercise history can be useful to build a tailored and feasible program (19,34). Several and validate tools for initial assessment are available for the exercise physiologist or kinesiologist to achieve this phase. For example, the physical activity readiness questionnaire can help define an initial risk profile of the subject (35), whereas the European Organization for Research and Treatment of Cancer QLQ-C30 can measure the quality of life and the severity of some symptoms and treatment-related side effects (36).

Paradoxically, the social distancing period may be a good time to start an exercise program, because some barriers that usually interfere with an active lifestyle adoption (eg, distance from gym, lack of time, traffic, and fixed time for lessons) are missing. Setting goals and tracking progress (Figure 1) using different instruments (eg, wearable technology and/or a personalized diary) can be an ideal strategy to stimulate patients to maintain adherence to the prescribed exercise program (34).

Goals should be established with the subjects, according to the following characteristics: specificity, measurability, achievability, realistic goals, and time availability. Cancer patients have unique needs related to their disease, therefore they should take all of their needs into consideration when choosing the most appropriate goals (eg, symptom control, improving mood, bodyweight, increasing exercise level), selecting those that are remotely assessable and most important for the patient. Moreover, the kinesiologist or physiotherapist should help the patients identify those exercise-related goals that are realistic and achievable. This aspect is crucial because failure in achieving the proposed goals can be extremely demotivating, particularly for oncological patients, with the possible consequence of exercise program dropout. Finally, goals should be time-based, remembering that the exercise prescription objectives may be influenced by the change in disease and treatment-related toxicities over time.

Another component that should be included in a home-based program, especially during the COVID-19 pandemic period, is the periodic follow-up (Figure 1) (34). This is important to maintain high engagement (37) and can be delivered by several modalities (eg, telephone, video chat platforms [ie, Skype], or email). The aims of the follow-up can be various: educate subjects to manage the exercise training, supervise the program, support patients to maintain an active lifestyle stimulating their motivation, reassess the situation, and modify the prescription. Follow-up time depends on several factors, such as the modality to deliver the program and the patients’ needs. These revaluations could be performed within a short interval at the beginning of the program to maximize the patient’s support and a longer interval later to favor the subject’s autonomy.

The exercise-program components should reflect guidelines, including type, frequency, duration, and intensity of the activities (19). Aerobic and strength exercises should be a key component of the exercise prescription, and their balance should be determined according to the patient’s goals and needs. Whereas strength activities require small spaces and limited equipment (eg, elastic bands, bodyweight exercises), the aerobic exercises could be difficult to include in a home-based program. If it is not possible to get outside or if the patient does not have access to a specific machine (eg, treadmill or cycle ergometer), a valid alternative could be represented by adapted exercises such as dancing or walking up and down the stairs. Moreover, the program should also include flexibility and proprioceptive training, especially for patients with specific symptoms or treatment-related side effects. Proprioceptive exercises could improve the chemotherapy-induced neuropathy, ameliorating the balance control, whereas regaining the joint range of motion through flexibility activities could be beneficial for patients undergoing surgery and presenting a limited range of joint extension.

In the home-based program, patients must be educated to self-monitor exercise intensity because, even if low-intensity may be appropriate for deconditioned patients, in other cases it may be insufficient to modulate the body homeostasis and increase the functionality, while high intensity may be unsafe. Thus, it is important to educate the patients to understand the intensity level using some practical tools, such as the heart rate or the perceived exertion scale.

It is also essential to define frequency (ie, the number of sessions per week, duration, and the time or sets, or repetitions per session or activity). Although the ACSM guidelines suggest a frequency of 2-3 times per week of 90 minutes of aerobic activities and 2 sets of 8-15 repetitions for strength training, it may be necessary to adapt these parameters to a particular patient’s clinical situation and disease. During the quarantine, patients have more free time to spend on exercising, but they may be sedentary or deconditioned, thus increasing the frequency and diminishing the duration may be a strategy to adopt. Nevertheless, it is necessary to remember that the “dosage” of exercise, in terms of type, frequency, duration, and intensity recommended by ACSM, may be not appropriate at the beginning for cancer patients and should be progressively reached, balancing the exercise-prescription components with the patient’s capacity.

Taking all these factors into consideration may allow the development of effective tailored exercise programs during COVID-19, which can be potentially carried on beyond the quarantine period to reduce the negative effect of sedentary behaviour, increase benefits related to PA and exercise, and ameliorate the psychological impairment due to the isolation and the outbreak emergency.

## Conclusion

The COVID-19 outbreak is a major challenge for global public health. Until a vaccine or specific therapies against COVID-19 are available, physical distancing and homestay remain the most effective approaches to slow down the spread of the infection. Nevertheless, these restrictive measures may decrease PA levels in cancer patients, with consequent deleterious long-term outcomes. In this light, promoting a remote, home-based lifestyle intervention in the cancer populations is an urgency, because if social distancing is necessary to stay healthy today, the physical inactivity that may be experienced will have negative and lethal effects tomorrow, especially in cancer patients.

## Funding

This research did not receive any specific grant from funding agencies in the public, commercial, or not-for-profit sectors.

## Notes

**Disclosures:** MM reports personal fees from Pfizer, EUSA Pharma, and Astra Zeneca. SP received honoraria or speakers’ fee from Astra-Zeneca, Eli-Lilly, BMS, Boehringer Ingelheim, Merck & Co., Roche, and Istituto Gentili. JW is a co-author of the American College of Sports Medicine exercise oncology guidelines. All remaining authors have declared no conflicts of interest.

**Role of the authors:** Alice Avancini and Sara Pilotto were involved in the study conception and design. Alice Avancini and Sara Pilotto wrote the initial draft of the manuscript and had the final responsibility for the decision to submit for publication. All authors participated in the drafting, reviewing, and approval of the final manuscript.

**Acknowledgments:** SP is supported by the Italian Association for Cancer Research AIRC-IG 20583 and was supported by the International Association for Lung Cancer (IASLC).

## Data availability

No new data were generated or analyzed in support of this research.
